# 2480. A *Legionella pneumophila* Investigation at an Outpatient Residence in a Tertiary Cancer Center

**DOI:** 10.1093/ofid/ofad500.2098

**Published:** 2023-11-27

**Authors:** Stephanie Rizzo, Rajkiran Kullar, Michell Reyes, Tania N Bubb, Anoshe Aslam

**Affiliations:** Memorial Sloan Kettering Cancer Center, Thornwood, New York; Memorial Sloan Kettering Cancer Center, Thornwood, New York; Memorial Sloan Kettering Cancer Center, Thornwood, New York; MSKCC, New York, New York; Memorial Sloan Kettering Cancer Center, Thornwood, New York

## Abstract

**Background:**

*Legionella pneumophila (L. pneumophila)* infections can severely affect immunocompromised patients. Found in water systems, the presence of *Legionella* in healthcare water sources creates an opportunity for hospital acquired infections. After a high-risk patient acquired *L. pneumophila* in June 2022 while at Memorial Sloan Kettering (MSK), a tertiary cancer center, Infection Control worked to identify the source. A timeline of events is illustrated in Figure 1. Between inpatient admissions, the patient stayed at MSK’s outpatient residence known as the Bone Marrow Transplant Residence (BMTR).

**Figure:**
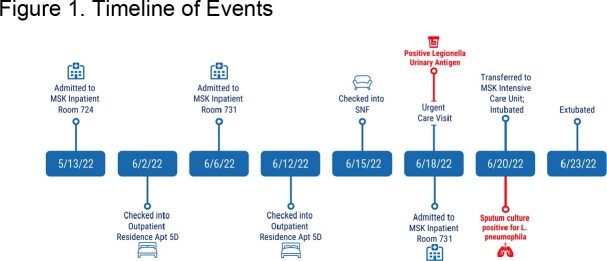

**Methods:**

Monthly surveillance cultures are collected at BMTR following a 2021 *L. pneumophila* investigation. To date, all surveillance cultures for *Legionella* were negative. Table 1 shows *L. pneumophila* patient cases at MSK from 2019 to 2022. Targeted environmental cultures were collected from inpatient rooms and the BMTR apartment. Inpatient cultures were negative but the apartment shower tested positive for *L. pneumophila* along with a respiratory culture from the patient. Both positive cultures were sent to a third-party lab for whole genome sequencing (WGS). Mitigation included chlorinated cleaning of showerheads in all BMTR apartments. Infection Control collected 93 environmental samples from all BMTR showers and kitchen sinks. To identify further clinical cases, surveillance was deployed on 53 patient admissions from BMTR to MSK between June and September. Upon admission, urine antigen tests were collected on patients who reported respiratory symptoms and resided at BMTR 21 days prior to presentation at MSK.

**Table 1.**
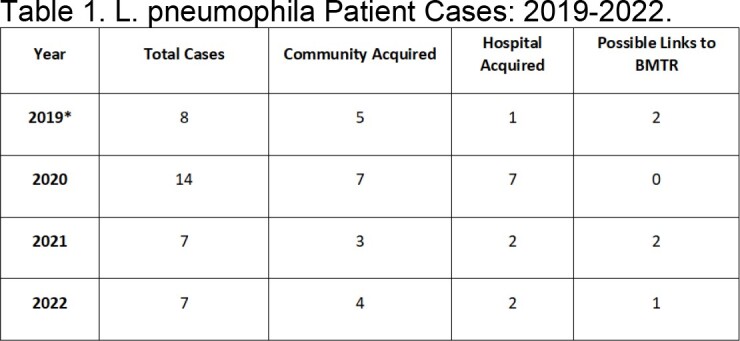
L. pneumophila Patient Cases: 2019-2022. *It should be noted that the two cases listed in 2019 were investigated but found not to be definitively linked to BMTR.

**Results:**

All 93 cultures were negative, and the patient and environmental isolates did not match from WGS. Of the 53 patients that were surveilled, 32 presented to MSK following or during their BMTR stays; 5 presenting with respiratory symptoms and zero testing positive for *Legionella*. The investigation was considered complete and closed.

**Conclusion:**

Although the isolates did not match, proactive surveillance emphasizes the importance of monitoring *Legionella* in outpatient settings. Monthly cultures and passive patient surveillance continues. Additionally, Copper-Silver ionization and UV disinfection and ultra-filtration are part of the water system at BMTR and monitored.

**Disclosures:**

**All Authors**: No reported disclosures

